# Prodrug Strategies for Enhancing the Percutaneous Absorption of Drugs

**DOI:** 10.3390/molecules191220780

**Published:** 2014-12-12

**Authors:** David D. N’Da

**Affiliations:** Centre of Excellence for Pharmaceutical Sciences (PHARMACEN), North-West University, Potchefstroom 2520, South Africa; E-Mail: david.nda@nwu.ac.za; Tel.: +27-18-299-2256; Fax: +27-18-299-4243

**Keywords:** prodrug, percutaneous absorption, enhancer, transdermal penetration

## Abstract

The transdermal application of drugs has attracted increasing interest over the last decade or so, due to the advantages it offers, compared to other delivery methods. The development of an efficient means of transdermal delivery can increase drug concentrations, while reducing their systemic distribution, thereby avoiding certain limitations of oral administration. The efficient barrier function of the skin, however, limits the use of most drugs as transdermal agents. This limitation has led to the development of various strategies to enhance drug-skin permeation, including the use of penetration enhancers. This method unfortunately has certain proven disadvantages, such as the increased absorption of unwanted components, besides the drug, which may induce skin damage and irritancy. The prodrug approach to increase the skin’s permeability to drugs represents a very promising alternative to penetration enhancers. The concept involves the chemical modification of a drug into a bioreversible entity that changes both its pharmaceutical and pharmacokinetic characteristics to enhance its delivery through the skin. In this review; we report on the* in vitro* attempts and successes over the last decade by using the prodrug strategy for the percutaneous delivery of pharmacological molecules.

## 1. Introduction

The skin, being the largest organ in the human body, has various functions, including protecting the body from its external environment, from excessive water loss, friction and impact wounds, and from external stimuli that could potentially harm the body, whilst also providing sensory abilities [1]. The skin of the average adult covers approximately 1–2 m^2^ [2] and receives a third of the total blood circulation, thus making it an ideal route through which therapeutic agents can be administered [3]. The outermost layer of the skin, the stratum corneum (SC), is the primary layer through which most drug molecules penetrate the skin. It is a compositionally and morphologically unique biomembrane that consists of a layer of compressed, keratin-filled corneocytes, anchored in a lipid matrix. This arrangement of the corneocytes and lipids makes the SC about a thousand times less permeable to water and substances, relative to other membranes in the body [2].

The advantages of the transdermal drug delivery (TDD), compared to the oral administration route, make it a very attractive alternative. Of these advantages include better patient collaboration, control over the input kinetics, with less side effects [2]. Incorporating drugs into a TDD system helps decrease their side effects on the gastrointestinal tract, whilst it can be especially advantageous to children or infants who have trouble taking some oral formulations, due to the bitter taste, or being unable to swallow tablets [4]. Since transdermal drug administration avoids the hepatic first-pass metabolism, an improved bioavailability would occur, the administered dose would be reduced and therapy could be easily interrupted in case of a toxic effect [5].

Despite these advantages, transdermal drug delivery (TDD) has its limitations, including: (a) the skin acting as a two-way barrier prevents molecules from entering the body from the external environment, while regulating the loss of essential body fluids and electrolytes; (b) drugs requiring high-blood levels can’t be administered through the skin; (c) patch adhesives may not adhere to all skin types and may result in allergic responses; and (d) drugs or drug formulations may cause skin irritation or sensitization.

Transdermal delivery is therefore unsuitable for all drugs. For molecules to pass through the SC, they need to exhibit favorable values with regards to certain physicochemical properties, including the partition coefficient (log P), melting point, aqueous solubility, and the molecular mass and pKa of the drug. All of these properties to various degrees influence the rate of a drug’s permeation through the skin [6].

Furthermore, physiological factors, such as the skin (age, hydration, condition and metabolism), body (anatomical) location, race, gender and temperature are reported to affect the rate of drugs’ permeation through the skin [7].

In this review, the structure of the skin, physiological and physicochemical factors governing percutaneous absorption of molecules and drugs, and representatives of different therapeutic categories of agents, subjected to the prodrug strategy over the last decade, are discussed.

## 2. Structure of the Skin

**Figure 1:**
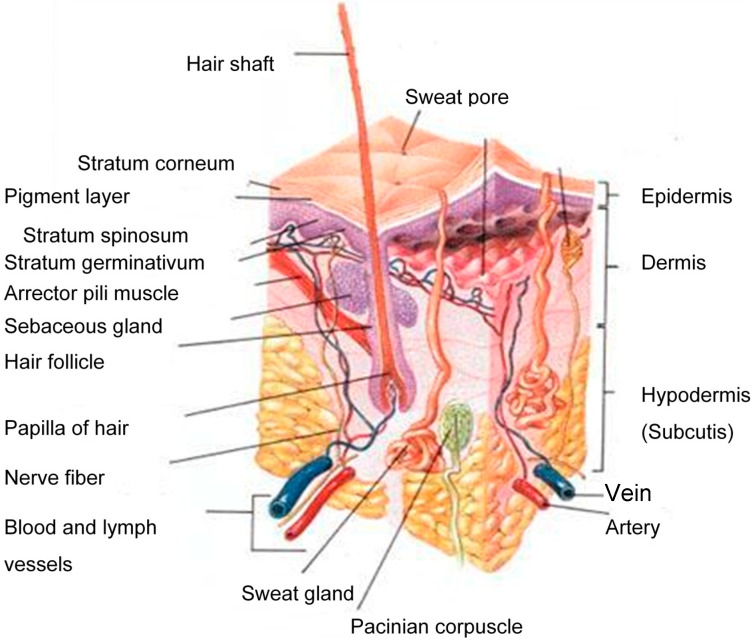
A schematic cross-section of human skin [9].

The human skin consists of three distinct layers,* i.e*., the stratified, avascular, cellular epidermis, the dermis and the underlying hypodermis (Figure 1) of connective tissue [8]. A therapeutic agent must cross all of these layers and enter the systemic circulation in order to exert its effect.

### 2.1. Stratum Corneum (SC)

The tissue that mainly affects drug permeation into the skin is the *stratum corneum* (SC), or the horny layer. The major components of the SC are the ceramides, fatty acids, cholesterol, cholesterol sulphate and sterol esters [10], which vary among individuals and body location [11] and provide the SC with its amphiphilic properties. Water plays a vital role in maintaining the SC integrity and is it also involved in the mediation of some hydrolytic enzymes’ activities.

### 2.2. Viable Epidermis

The epidermis is situated directly beneath the SC and is described as a complex, multiple-layered membrane. The epidermis contains no blood vessels, and must nutrients and waste products travel across this membrane in order to maintain its integrity.

### 2.3. Dermis

The dermis is situated between the epidermis and the underlying subcutaneous, fatty layer,* i.e*., the hypodermis. It is the major component of the skin and predominantly consists of collagen fibrils and elastic tissue, which provide support and flexibility to the skin. Since the dermis is very hydrophilic, hydrophilic drugs would easily pass through it, while lipophilic drugs would experience difficulties in crossing it. Structures, like lymphatic and blood vessels, nerve endings, hair follicles, sebaceous and sweat glands are found in the dermis [12]. The dermis layer has the highest blood circulation in the skin and thus regulates body temperature and removes toxins and other undesirable molecules. The dermis thus forms a “sink” condition that allows transdermal permeation to take place, due to the formed concentration gradient [13].

### 2.4. Hypodermis

The hypodermis, also known as the subcutaneous fatty layer, bridges the overlying dermis and the underlying body constituents. This layer is very thick and thus insolates the body and protects it against external shock.

### 2.5. Skin Appendages

Three main appendages are present in the skin, namely the hair follicles, and the eccrine and apocrine sweat glands. The hair follicles are found over the entire body, except in areas that take a lot of strain, such as the feet soles and the hand palms. All three appendages are situated in the dermis [14] and each has specific functions. The sebaceous glands are located in the hair follicles and secrete sebum, a mixture of fatty acids, cholesterol, triglycerides, waxes and cellular debris [15]. More lipophilic drugs hence cross this section, due to the lipophilic nature of sebum. Eccrine glands are found across most of the body and secrete sweat, a diluted salt solution with a pH ~5, and thus function as body heat regulators. The apocrine glands are located at specific areas of the body only and are usually limited to the axillae, nipples and anogenital regions, and are responsible for the odor of sweat [7].

## 3. Routes of Transdermal Delivery

**Figure 2:**
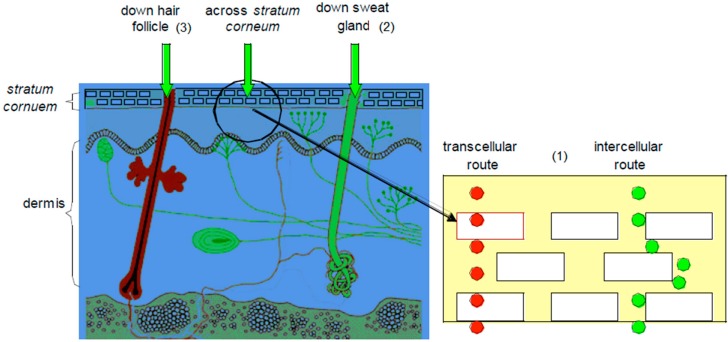
A representation of transdermal delivery pathways: (**1**) transepidermal route (intra- and intercellular); (**2**) the sweat gland and (**3**) the hair follicle (adapted from [19]).

For molecules to affect the body, they must cross through the different layers of the skin to reach the systemic circulation. This can be achieved through different routes,* i.e*., the transepidermal routes (intra- and intercellular), the sweat glands and the hair follicles [1,7], as illustrated in Figure 2.

### 3.1. Transepidermal Routes

This pathway is split into the intracellular (crossing of molecule through the cells) and the intercellular (crossing of molecule in between the cells) routes.

*The intracellular route*, also known as the transcellular pathway, is the path through which a molecule permeates through the SC. Drug molecules that use this route move across and through the corneocytes located in the SC [1]. The keratinocytes of the SC are very hydrophilic in nature, and thus naturally, hydrophilic drugs would cross the SC with more ease. These hydrophilic keratinocytes are, however, enclosed in a multi-layered lipophilic membrane and are surrounded by a lipophilic matrix in between these cells. This lipophilic environment thus prevents hydrophilic drugs from crossing the SC [7].

*The intercellular route* accommodates molecules that pass in between the cells of the SC. The lipid bilayers, comprising up to 1% of the SC, are important in reducing water loss through the skin. The balance of lipid and aqueous solubility that is important for solubility in the membrane is also important for molecules traversing the intercellular route [16,17,18].

### 3.2. Transappendageal (Shunt Routes)

The sweat glands and hair follicles also offer pathways through which molecules can travel through the SC. Drugs that use this route normally have high molecular weights and do these charged molecules travel through the bulk of the SC with difficulty [7].

## 4. Factors Influencing Skin Permeation of Drugs

The transdermal permeation of drugs is significantly affected by both physiological and physicochemical factors, as discussed in the following sections.

### 4.1. Physiological Factors

The physiological factors that influence the absorption of molecules through a healthy skin include:

#### 4.1.1. Skin Age

As the skin ages, some functional and structural changes take place that affect the transdermal absorption of molecules into it. Hydration plays an important role in transdermal absorption and decreases with age, as the skin loses its moisture content [20]. The enzymatic activity in the skin also reduces with age, as well as the blood flow to the skin. A lower blood flow in older people suggests that the clearance of drugs in an older SC will be slower, which in turn would negatively impact on the drug flux gradient [7].

#### 4.1.2. Anatomical Location

The skin differs in appearance all across the body. The generalized decreasing order of absorption exists from the genitals > head and neck > trunk > arm > leg [7]. Differences in anatomical sites are therefore indicative that the location of transdermal patches, or ointment applications would affect absorption.

#### 4.1.3. Race

Racial differences in skin function have been investigated. Weigand* et al*. [21] reported an increase in intracellular cohesion in black skin, while Reinertson and Wheatley [22] further demonstrated higher lipid content in black skin. Although relatively limited data are available on race, indications are that racial differences do exist among white and black skins, with regards to some anatomical and physiological functions of the skin, and that these differences could determine race related patterns in skin behavior, namely the response to irritant chemicals and the penetration of topically applied drugs.

#### 4.1.4. Temperature

The permeation of molecules across the skin is a passive process. An increase in temperature would thus result in an increase in kinetic energy of the drug molecules, which would therefore cause the molecules to move faster through the SC. An increasing temperature would also cause structural alterations in the SC and underlying tissue and result in a faster movement of the drug through the different skin layers [7,23].

#### 4.1.5. Skin Hydration

The water content of the skin plays a vital role in transdermal permeation. Jackson [24] found that hydration of the skin through soaking, moisturizing, or humidity, for example, causes the corneocytes in the SC to swell, which allows molecules to permeate through that layer much easier. In another study, Li and Chan [25] revealed that the use of a very hydrophilic co-solvent, like ethanol/water, had increased the permeation of anti-human immunodeficiency virus (HIV) drugs through the skin.

#### 4.1.6. Skin Condition (Disease)

Disease compromises the natural barrier function of the skin, which affects the transdermal absorption of therapeutic agents. The rate of transdermal absorption would increase with a disease that ravages the skin, but would decrease upon self-healing of the skin [7].

#### 4.1.7. Skin Metabolism

Enzymes in the skin are responsible for metabolism and for the elimination of drugs. For prodrugs, which have to undergo biotransformation, enzymatic activity is therefore a rate limiting factor in the transdermal absorption process [26], and is reported to be the highest in the viable epidermis of the skin [27].

### 4.2. Physicochemical Factors

Physicochemical factors are the primary factors that influence the transdermal absorption of drugs. For molecules designed to pass through the SC, favorable physicochemical properties are necessary [2], which include:

#### 4.2.1. Solubility

The SC is the most important barrier to skin permeation and therefore the rate limiting factor for drugs to cross the skin. The SC is a lipophilic membrane [28] and the amount of a drug that accumulates in it bears some relationship to the solubility of that drug in some organic solvents, such as the highly lipophilic hexane. Saturated solutions better permeate through the SC, because they represent maximum thermodynamic activity [29]. An awareness of the importance of drug solubility in the SC emerged from reports during the 1980s that drugs with biphasic (water and lipid) solubility better permeated than those with high monophasic (water or lipid) solubility [17,18]. A very hydrophilic drug is unable to penetrate the skin, while a very lipophilic drug has the propensity to remain in the layers of the SC. While the SC is lipophilic in nature and favors the permeation of lipophilic drugs, the aqueous nature of the layers beneath the SC dictate that drugs should embody some hydrophilic properties to pass through them. Often, small structural changes of a drug, such as salt formation, or esterification can either enable the enhancement of aqueous, or lipid solubility [30]. Moieties, such as polyethylene glycol (PEG) that are amphiphilic in nature, show an increase in the aqueous solubility of derivatives, due to multiple hydrogen bonds that form with water molecules, resulting from an increase in chain length [31].

#### 4.2.2. Partition Coefficient

Penetration of the SC requires that a drug partitions into the membrane. Such partitioning is an important step in the penetration of the membrane [32]. However, solubility in the membrane is the limiting physicochemical parameter. The partition coefficient is usually the key factor in determining which pathway a drug molecule would follow when passing through the SC. Hydrophilic molecules are therefore expected to pass through the intracellular routes, whereas more lipophilic molecules would use the intercellular routes. Either lipophilic or hydrophilic permeants must pass through the hydrophilic and lipophilic layers, respectively, to achieve successful transdermal delivery. In order to study and determine the partition coefficient of a drug, organic solvents are used to mimic the different phases/domains that a molecule must cross to enter the systemic circulation. The *n*-octanol-water partition coefficient (log P) is a good representation of the partitioning of a drug in between the lipophilic SC and the underlying hydrophilic living cells of the epidermis [33]. The log D value (the log P value at a specific pH) of a compound is usually a good indication as to whether a molecule would be a favorable candidate for transdermal permeation, or not. The lower the log P value, the more hydrophilic is the compound. Compounds with a log P lower than −1 would hence have difficulty in passing from the vehicle into the SC. Subsequently, only compounds with log P > −1 should be considered as possible candidates for transdermal delivery. Another problem arises when compounds have a log *p* value above 2. Such a drug is delayed in the SC, where a reservoir could be established that would create problems with achieving steady plasma concentrations within a reasonable time span [16]. Williams [7], however, reported that molecules with a log *p* value in the 1–3 range, had exhibited both aqueous and lipophilic properties, sufficient enough to obtain proper transdermal permeation, because they had been able to cross the lipophilic (SC) and hydrophilic layers (epidermis) of the skin. Such findings emphasize the lack of consensus among researchers with regards to the optimum partition coefficient range that is required for a drug to be efficiently delivered across the skin.

#### 4.2.3. Molecular Mass

The skin is a compact membrane that is difficult to penetrate. Smaller molecules are therefore likely to permeate the skin at higher rates than larger molecules, hence the importance of molecular size in transdermal absorption. Idson [34] suggested an inverse relationship between molecular size and transdermal permeation. Drugs with a molecular mass (MW) between 100 and 500 Daltons were found to be suitable for transdermal transport [2,7]. A study conducted on a molecule with MW above 500 Daltons; however, also resulted in percutaneous absorption [35], suggesting that the upper limit of the MW range of a compound could indeed exceed 500 Daltons, but still efficiently permeate the skin through passive diffusion.

#### 4.2.4. Ionization

The lipophilic nature of the SC had led to the belief that ionized drugs would be poor candidates for transdermal delivery. As a result of the complex structure of the skin, however, drugs can cross the skin via various pathways. The transcellular route is regarded as having intermediate properties, whereas the intracellular route is mainly regarded for allowing the delivery of lipophilic molecules. Ionized drugs hence cross the skin through the shunt route, but the amount of molecules that pass through that route is significantly less than unionized molecules that take the intracellular route [7].

The pH range of the viable epidermis is 7.3–7.4 and that of the SC 4.2–5.6. The drug concentration that exists in the unionized form is a function of both the dissociation constant of the drug and the pH at the absorption site [30].

From the Henderson-Hasselbach equation:

(1) $$\text{Aci drug:pH}=\text{pKa}+\text{log}\frac{\left(\text{salt})(\text{ionized}\right)}{\left(\text{acid})(\text{unionized}\right)}$$

(2) $$\text{Base drug pH}=\text{pKb}+\text{log}\frac{\left(\text{salt})(\text{ionized}\right)}{\left(\text{acid})(\text{unionized}\right)}$$

The fraction of the unionized drug is thus a function of the pH [36].

#### 4.2.5. Hydrogen Bonding

The varied skin components (lipids, proteins, aqueous regions, enzymes,* etc.*) and possible drug penetrants (weak acids/bases, ionized/unionized species, neutral molecules,* etc.*) suggest a multitude of potential interactions between drug substances and skin tissue [7]. The formation of hydrogen bonds, or weak Van der Waals forces, would hence influence skin penetration of a permeating drug. Lag time is an important factor being influenced by drug binding to the tissue. Bound drugs to skin tissue in the SC would result in a prolonged lag time, which would translate into a delayed onset of the therapeutic action. Since the SC is mainly a hydrogen bond acceptor [37], an increase in the number of hydrogen bonding groups of the drug may inhibit its permeation across the layers of the SC [7].

#### 4.2.6. Melting Point

Generally, organic substances with high melting points and high enthalpy of melting have lower aqueous solubility properties, because solvents can’t enter the crystalline structure of such molecules to dissolve them [38]. An indirect relationship therefore exists between the melting point and the solubility of a drug. Hadgraft* et al.* [39] developed a model that accurately estimates the solubility constraint (δ_SC_) in the SC by using the melting point of the drug:

(3) $$\text{Log}\delta \text{sc}=1.911\frac{10^{3}}{\text{mp}}-2.956$$

where, δ_SC_ is the solubility constraint in the SC, mp is the melting point (in K).

Lowering the melting point of a drug would hence cause an increase in its solubility in the SC and ultimately in its permeation across the skin [40].

## 5. The Impact of Prodrug Strategy on the Percutaneous Absorption of Drugs

Physicochemical properties, like the lipophilicity of a drug molecule can be modified through derivatization. The fact that most drugs are only designed for oral administration emphasizes the complexity of the development of drug candidates suitable for transdermal delivery, because their physicochemical makeup has to be specifically modified to allow skin permeation. The derivatization of a drug may enhance its ability to permeate through the skin. This approach comprises the basis of the prodrug strategy, during which a drug molecule is structurally modified into derivatives, imparted with favorable physicochemical properties to facilitate its permeation, although being designed to latter yield the parent drug (active pharmaceutical ingredient) after enzymatic, or chemical release [41]. Two of the derivatization paths often used include (a) increasing lipophilicity through alkyl-chain lengthening [42], and (b) using PEGylation to bring about derivatives, such as esters, carbonates, carbamates and ether, equipped with enhanced physicochemical properties that would more readily cross the skin, compared to the parent drug [43]. This strategy could lead to an increase in a drug’s pharmacological action in the body. Several drugs, representing different categories of therapeutic agents, have been investigated over the last decade for their skin permeation ability, by employing the prodrug strategy. Typical examples of such drugs are discussed in the following sections.

### 5.1. Nucleoside Anti-Retrovirals (ARVs)

Acquired immune deficiency syndrome (AIDS) is one of the deadliest infectious diseases worldwide and mainly affects populations in developing countries. Globally, an estimated 35.3 million people were living with HIV in 2012, an increase from previous years, measured by the fact that more people were receiving the anti-retroviral therapy. Two millions and three hundred thousands new HIV infections were reported globally in 2012, showing a 33% decline in the number of new infections from 3.4 million in 2001. Furthermore, the number of AIDS deaths had also declined from 2.3 million in 2005 to 1.6 million in 2012 [44]. The number of AIDS deaths, however, by far exceeded those from any other infectious human disease. HIV and AIDS therefore remain the focus of global health and socio-economic concerns today.

Anti-retrovirals (ARVs) are “cocktails” of nucleoside drugs, aimed at slowing down the progress of the infection so as to avoid development into the AIDS disease, or to prevent transmission of the virus from pregnant women to unborn babies. ARV drugs, including zidovudine (AZT), lamivudine (3TC), stavudine (d4T), didanosine (DDI), zalcitabine (DDC) and others, are administered orally, while daily repetitive doses must be taken to sustain therapeutic levels of plasma concentrations, resulting in high toxicity. The transdermal administration of ARVs as an alternative route has been attracting increased interest, because of the expectation of better patient compliance that would, concurrent to improved life quality, also result in a decrease in resistance to ARVs. Patient compliance especially in pediatric patients is expected to be improved, due to avoiding the poor taste of current oral ARV medication.

Despite all of the advantages of this pathway, the skin’s barrier effect limits this route to drugs that possess specific physicochemical properties [2]. These limitations have forced researchers to investigate alternative strategies for enhancing the transdermal permeation of drugs through the skin. Some of these strategies include the development of derivatives as prodrugs, by using penetration enhancers and by incorporating transport mechanisms (ethosomes and liposomes) to enhance transdermal drug permeation. The inclusion of an enhancer within a formulation may, however, also increase the absorption of undesirable components, which can cause skin damage and irritancy. A promising alternative to using penetration enhancers is the prodrug approach [18,41]. This involves modifying the chemical structure of a drug with concomitant changes in both its physicochemical and pharmacokinetic characteristics, to enhance its delivery.

In adopting the prodrug strategy, N’Da* et al*., synthesized a series of methoxypolyethylene glycol (MPEG) carbonate derivatives of AZT **1**. The aqueous and lipid solubility within this series of prodrugs/derivatives were found to increase as the chain lengthened, which was in accordance with the amphiphilic nature of PEG. The potential of the series of derivatives to penetrate the skin was evaluated* in vitro* by measuring their passive diffusion through excised human female abdominal skin during Franz cell diffusion studies in saturated PBS pH 7.4 solutions. Prodrug **2** with eight ethylene oxide (EO) units was the most effective penetrant, permeating the skin with a flux of 53.3 ± 46.5 nmol/cm^2^/h, which was 2.4–10.1 times higher than that of AZT (8.55 ± 5.3 nmol/cm^2^/h) [45].

In a similar study, using stavudine **3** instead, the most effective penetrant was derivative **4** with three EO units in the side chain, exhibiting a flux of 26.1 nmol/cm^2^/h compared to 59.15 nmol/cm^2^/h of the parent drug. No skin permeation enhancement was therefore observed for stavudine [46].

Gerber* et al*. [47] investigated the skin permeation potential of lamivudine **5** in a study of *n*-dialkyl ester/amide prodrugs from saturated PBS (pH 7.4) solutions. The best penetrant was *n*-propyl derivative **6**, with a median flux of 0.046 µmol/cm^2^/h; far lower than 4.3 µmol/cm^2^/h of lamivudine. Thus, no skin permeation enhancement was achieved during this study by employing the prodrug strategy.

**Figure 3:**
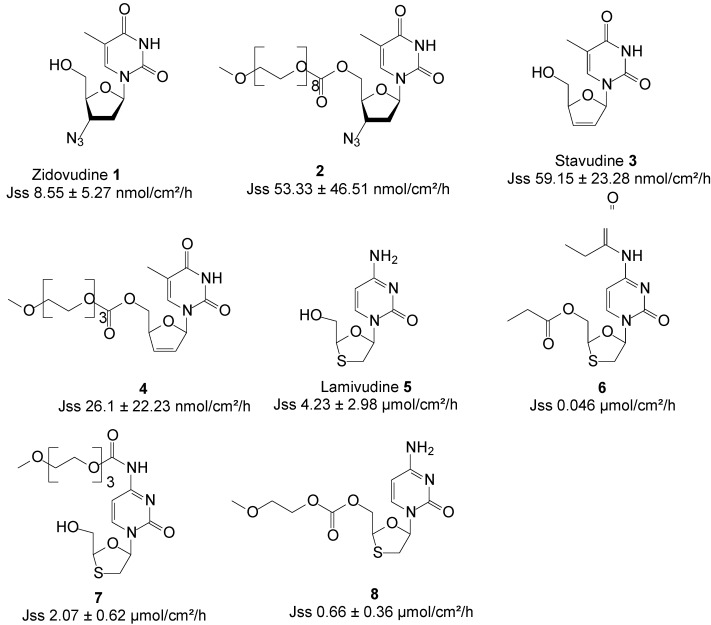
Structures of ARV drugs and best skin penetrant derivatives.

Van Heerden *et al*. also investigated the skin permeation of oligomeric MPEG carbonate and carbamate prodrugs of lamivudine from saturated PBS solutions as did earlier Gerber and co-workers. Carbamate **7** and carbonate **8**, featuring one and two EO units, respectively, were the best permeants with steady-state flux (J_ss_) values of 2.06 and 0.66 µmol/cm^2^/h, respectively [48]. These flux values were lower than 4.23 µmol/cm^2^/h of lamivudine. The prodrug strategy also failed to enhance skin permeation in this study. The structure of the parent drugs and derivatives are illustrated in Figure 3.

### 5.2. Cytarabine (Anticancer)

**Figure 4:**
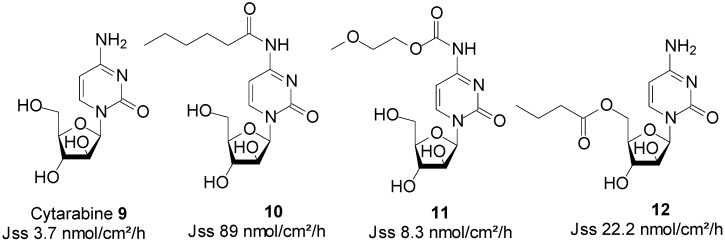
Cytarabine and its best skin permeant derivatives.

Cytarabine (Figure 4) is an anticancer drug that is extensively used in the treatment of both acute and chronic myeloblastic leukaemia. The major impediments to a broad clinical use of cytarabine include its cell cycle (S-phase) specificity, and the rapid metabolism of the drug in plasma into its inactive metabolite, uracil arabinoside (ara-U), by the enzyme deoxycytidine deaminase [49]. A prolonged exposure of cells to cytarabine’s cytotoxic concentrations is a requirement for achieving maximum activity. In practice, it is administered either through repetitive schedules, or continuous intravenous infusion to achieve a sustained supply [50]. Patient non-compliance occurs as a result of these modes of administration being inconvenient and invasive.

In search for a viable alternative to the intravenous route of administration of cytarabine, Legoabe* et al*. investigated the skin permeation ability of cytarabine **9**, by employing the prodrug strategy. All Franz cell diffusion experiments were performed at saturation in aqueous PBS pH 7.4 solutions. They observed an increase in skin permeation fluxes by their series of N^4^-alkylamide derivatives. *N-*Hexyl amide **10** was found to be the best penetrant with a J_ss_ value of 89.0 nmol/cm^2^/h, which was significantly higher than 3.7 nmol/cm^2^/h of cytarabine [51]. In another study, the *n*-alkyl chains were replaced by MPEG moieties, which resulted in a series of N^4^-MPEG carbamates. Although not significantly higher, skin penetration was enhanced, since the J_ss_ of carbamate **11**, featuring one EO, was found to be twice as high than that of cytarabine (8.3* vs.* 3.7 nmol/cm^2^/h) [52]. The study of *N*-alkyl ester prodrugs of cytarabine also led to enhanced skin permeation. Indeed, *N*-butyl ester **12** exhibited the highest flux value of 22.2 nmol/cm^2^/h, which was significantly higher than that of the parent drug cytarabine (3.70 nmol/cm^2^/h) [53]. The structures of cytarabine and its best permeant derivatives are shown in Figure 4.

### 5.3. Morphine (Opioid Analgesic)

Morphine **13** is the most widely used opioid analgesic for acute and chronic pain, and is the standard against which new analgesics are measured. Morphine is inefficiently absorbed when orally administered, due to first-pass metabolism. In patients with normal renal function, the plasma half-life of morphine is 1.4~3.4 h [54].

**Figure 5:**
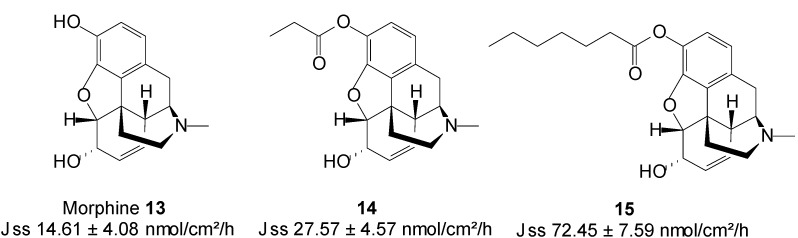
Morphine and its *n*-alkyl ester prodrugs.

Transdermal administration would offer a further improvement in its delivery, by enabling the continuous systemic application of morphine through intact skin, hence producing constant plasma concentrations [55]. Two *N*-alkyl esters (Figure 5) of morphine, *viz*. morphine propionate **14** and morphine enanthate **15** were synthesized as potential prodrugs for transdermal delivery. Both prodrugs were more lipophilic than their parent drug. The* in vitro* skin permeation of morphine and its prodrugs from citrate-phosphate buffer in pH 6 was in the order of **15** > **14** > **13**. Morphine propionate and morphine enanthate therefore enhanced the transdermal delivery of morphine by 2- and 5-fold, respectively [56]. The highest flux observed with the emanate prodrug may be ascribed to the fact this compound was assessed in saturated conditions unlike the propionate **14** which was tested in dilution.

### 5.4. Naltrexone (Opioid Antagonist)

Naltrexone (NTX), an opioid antagonist, is used for alcohol dependence and opioid addiction [57]. In maintenance therapy for alcohol abstinence, the NTX dose must be given regularly to maintain a minimum effective plasma drug concentration. Standard drug regimens for chronic therapy create non-compliance problems and require high motivation of NTX patients [58]. Sustained release formulations would be extremely useful in maintenance therapy and may result in the improvement of the therapeutic effectiveness of NTX. Additionally, a non-oral delivery route for NTX could reduce oral NTX related gastrointestinal side effects, including nausea, abdominal pain and vomiting [41,59]. NTX bioavailability would also be increased via a delivery route that bypasses first-pass metabolism, as this drug is found to undergo extensive first-pass metabolism with a resultant low oral bioavailability of a mere 5%–40% [60].

In search for a non-oral delivery route for NTX **16**, Stinchcomb and co-workers assessed the human skin permeation of straight-chain alkyl ester, and branched-chain alkyl ester and carbonate prodrugs of naltrexone alongside the parent drug from mineral oil in saturated conditions. All the straight-chain ester prodrugs enhanced the skin permeation of NTX. The acetate **17** and propionate **18** were the best permeants with J_max_ values 15.6 ± 6.3 and 11.1 ± 3.7 nmol/cm^2^/h, respectively, higher than 2.5 ± 1.5 nmol/cm^2^/h of NTX [61].

**Figure 6:**
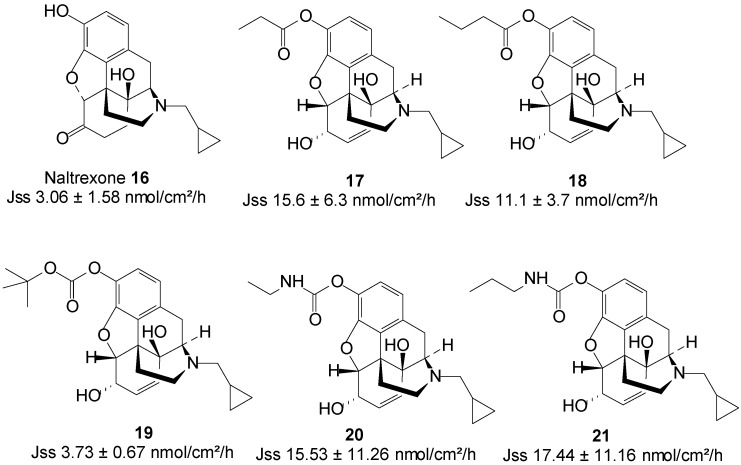
Naltrexone and its best skin penetrant prodrugs.

Outcomes of branched-chain prodrugs were disappointing as most derivatives displayed significantly lower transdermal fluxes than parent NTX. The best penetrant was found to be *tert*-butyl carbonate **19** (Figure 6), having a steady-state flux (J_ss_) value of 3.73 ± 0.67, which was similar to 3.06 ± 1.58 nmol/cm^2^/h of the parent NTX [62]. Thus, no skin permeation enhancement was achieved in this study.

Guided by the findings of previous studies, they further investigated the human skin permeation of 3-*O*-alkyl carbamate prodrugs of NTX. Two series of prodrugs viz. straight-chain *N*-monoalkyl and branched-chain *N,N*-dialkyl carbamates (Figure 6) were synthesized. Here again, all the straight-chain carbamate prodrugs enhanced the skin permeation of NTX while the branched-chain derivatives did not. Interestingly, like in the case of the straight-chain esters, the *N*-ethyl **20** and *N*-propyl **21** carbamate prodrugs were the best penetrants, enhancing the permeation of NTX up to 3-fold [63]. The results of these three studies confirm that the nature and type of promoiety are crucial to obtaining maximum skin permeation of a drug using the prodrug strategy.

### 5.5. Non-Steroidal Anti-Inflammatory Drugs (NSAIDs)

Non-steroidal anti-inflammatory drugs (NSAIDs) are also known as non-steroidal anti-inflammatory agents/analgesics (NSAIAs), or non-steroidal anti-inflammatory medicines (NSAIMs). This group of medications has analgesic (pain reducing) and antipyretic (fever reducing) effects. In higher doses, they also have anti-inflammatory effects by reducing swelling.

Ketorolac is an NSAID agent with powerful analgesic and low anti-inflammatory activities, and is widely used in the management of both moderate and severe pain [64]. Although the oral bioavailability of ketorolac is reported to be 90%, with very low hepatic first-pass elimination, its biological half-life of 4–6 h requires frequent administration to maintain a therapeutic effect [65]. The long-term use of currently available dosage forms of ketorolac results in gastrointestinal ulceration and acute renal failure [66]. The transdermal delivery route is regarded as a viable alternative for the safe delivery of ketorolac.

**Figure 7:**
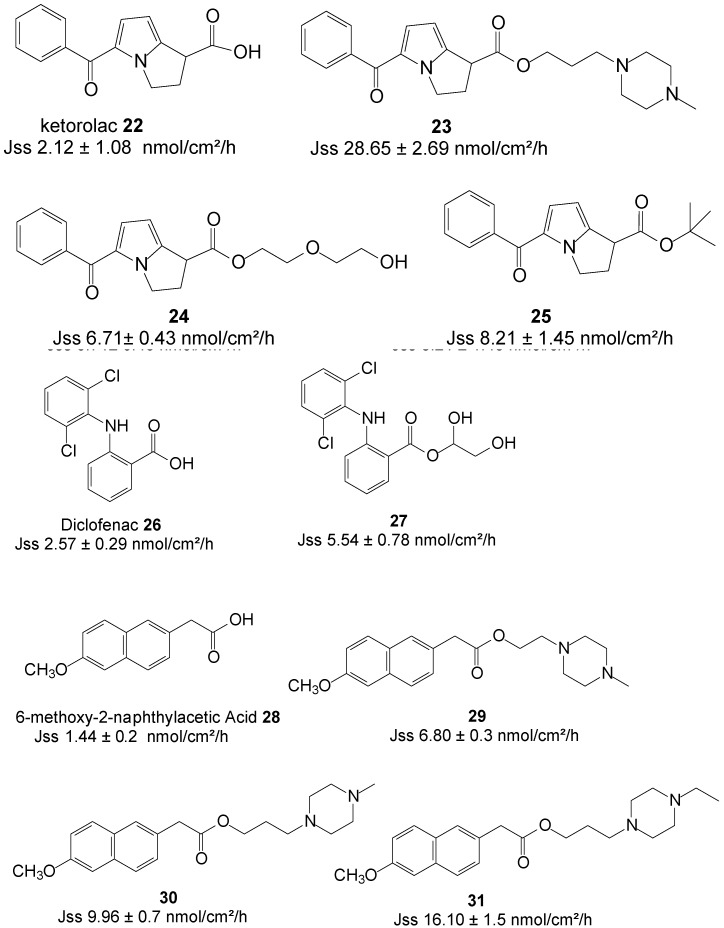
Structures of NSAIDs and their best permeant ester prodrugs.

Quandil* et al.* [67] synthesized a series of piperazinylalkyl ester prodrugs of ketorolac **22** and assessed their potential for permeating dorsal rat skin alongside the parent drug in PBS. The study resulted in a significant increase in skin permeation of the prodrugs. The best permeation rate was achieved with ester **23** (Figure 7) with J_ss_ values of 44.60 ± 12.14 nmol/cm^2^/h and 24.15 ± 3.50 nmol/cm^2^/h at pH 5 and 7.4, respectively. These values were significantly higher than those ketorolac; 28.65 ± 2.69 and 2.12 ± 1.08 nmol/cm^2^/h at pH 5 and 7.4, respectively. Skin permeation was thus enhanced in the study.

Polyoxyethylene glycol ester prodrugs of ketorolac were also synthesized and their ability to permeate human skin* in vitro* was also assessed. Gel B with the composition of carbomer (1%), triethanolamine (1%), distilled water (83%) and the ester prodrug **24** (15%) (Figure 7), featuring two EO units, was found the best permeant with a J_ss_ value of 6.71 ± 0.43 nmol/cm^2^/h, slightly higher than 4.1 nmol/cm^2^/h of gel A containing ketorolac (15%) instead [68].

More recently, Liu* et al*. investigated the impact of ester promoieties on the transdermal delivery of ketorolac. Different *n*-alkyl and aryl ester prodrugs of ketorolac were synthesized and their permeation across nude mouse skin evaluated in a 30% ethanol/pH 7.4 Buffer. Ketorolac *tert*-butyl ester **25** (Figure 7) exhibited the highest penetration of all compounds and tested with a flux of 8.21 ± 1.45 nmol/cm^2^/h, which represented an enhancement ratio of 2.5, compared to ketorolac (3.24 ± 0.25 nmol/cm^2^/h) [69].

The transdermal delivery of diclofenac **26** was also evaluated by using the prodrug strategy. Methanol, ethylene glycol, glycerol and 1,3-propylene glycol esters were synthesized and the *In vitro* fluxes across human epidermal membrane determined using saturated aqueous solutions (vehicle, PBS pH 7.4). The glycerol ester prodrug **27** was the best permeant of all with a flux of 5.54 ± 0.78 nmol/cm^2^/h, which was almost 2-fold higher that of diclofenac itself (2.57 ± 0.29 nmol/cm^2^/h) [70]. Diclofenac displayed higher permeation flux across hairless rat skin than its prodrugs from hydroxy-functionalized polyacrylate patches. This inferiority in fluxes of the prodrugs has been ascribed as probably due to strong interaction between the ester prodrugs and the ester type adhesives [71].

Furthermore, another NSAID which has recently been investigated is nabumetone. Piperazinylalkyl ester prodrugs (Figure 7) of its active metabolite, 6-methoxy-2-naphthylacetic acid **28** were synthesized and evaluated* in vitro* from saturated PBS solutions for the purpose of percutaneous drug delivery. Rat dorsal skin was used in Franz diffusion cells. The 4-ethylpiperazinyl-1-propyl ester prodrug **31** showed a 10-fold higher flux in permeating the skin compared to the parent **28** (16.10* vs.* 1.44 nmol/cm^2^/h) [72]. Thus, enhancement of percutaneous absorption was achieved in this study by using the prodrug strategy.

### 5.6. Apomorphine (Anti-Parkinsonian)

Apomorphine **32** is a classic mixed type of dopamine D1 and D2 receptor agonists, used in the therapy of Parkinson’s disease [73]. Orally administered apomorphine in advanced Parkinson’s disease patients is unsuccessful, due to its high dose requirement as a result of metabolic constraints and the first-pass metabolic effect. High oral doses of apomorphine can furthermore cause gastrointestinal complications, associated with nephrotoxicity [74]. Apomorphine is most commonly administered through repeated subcutaneous infusions or injections, which invariably result in the appearance of subcutaneous nodules. The transdermal delivery of apomorphine could be an ideal alternative route of administration, due to the advantages of eliminating the first-pass metabolism, a reduction in gastrointestinal side effects, and sustained release to resolve its short half-life (32 min).

**Figure 8:**
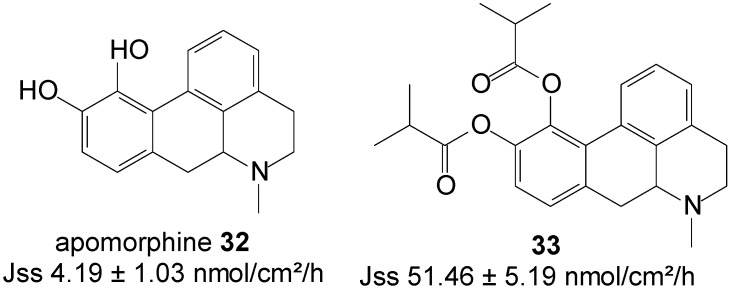
Apomorphine, its diester prodrug, and their respective skin permeation fluxes.

Diester prodrugs of apomorphine *viz.* diacetyl apomorphine and diisobutyryl apomorphine were synthesized and their* in vitro* permeation across nude mouse skin assessed from saturated deionized solutions. Significantly higher skin permeation was achieved during this study with diisobutyryl apomorphine ester **33** (Figure 8). This prodrug displayed a J_ss_ flux of 51.46 ± 5.19 nmol/cm^2^/h, which exceeded the flux of apomorphine base (4.19 ± 1.03 nmol/cm^2^/h) by 10-fold [75].

### 5.7. Bupropion (Anti-Depressant)

Bupropion (BUP) **34** is an aminoketone that is used as an antidepressant and non-nicotine aid to smoking cessation [76]. In addition to its side effects, such as nausea and vomiting, even cases of seizures, either due to BUP overdoses or unintentional exposure, are reported [77]. To curb the dose related risk of seizures, associated with the high peak concentration of the drug following oral administration, BUP HCl is administered in split doses. Alternatively, the transdermal delivery of BUP would reduce its dose related side effects, increase its bioavailability by reducing its metabolism and the first-pass metabolic effect, and allow for the easier removal of drug input, while maintaining therapeutic concentrations for long periods, with non-invasive zero-order release.

**Figure 9:**
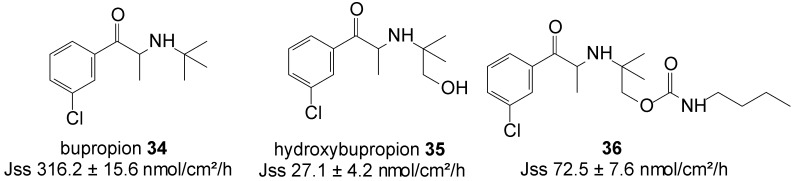
Bupropion, hydroxypropion and the carbamate prodrug of hydroxypropion.

Carbamate prodrug **36** of hydroxybupropion **35** (Figure 9) was synthesized and the* in vitro* percutaneous absorption through excised human skin assessed, alongside bupropion **34 **and **35** from saturated mineral oil. The* in vitro*, mean steady-state flux of **34** was significantly higher than that of hydroxybupropion **35** (316.2 ± 15.6 *vs*. 27 ± 4 nmol/cm^2^/h). Although bupropion traversed human skin at sufficient rates to achieve the required plasma levels, however, it was found chemically unstable and hygroscopic, and unsuitable for transdermal formulation. Contrary, although hydroxybupropion was found stable, its transport across skin was much slower. Alternatively, prodrug **36** was found to be stable, whilst also providing a 2.7-fold increase in the transdermal flux of hydroxybupropion **35** (72.5 ± 7.6* vs.* 27 ± 4 nmol/cm^2^/h) across human skin* in vitro*. Carbamate **36** was hence regarded as a viable option for the transdermal delivery of hydroxybupropion [78].

### 5.8. Theophylline (Cardiovascular)

Theophylline is a methylxanthine drug that is intravenously administered in therapies for respiratory diseases, such as asthma. The use of theophylline is complicated by its interaction with various drugs, mainly cimetidine and phenytoin. Because it has a narrow therapeutic index, it must be monitored through direct measurement of serum theophylline to avoid high levels related toxicity. Seizures can also occur in severe cases of toxicity. The topical delivery of theophylline would as a result appear as a credible route to reduce its risk of high dose related toxicity and other possible side effects, including nausea, diarrhea, increased heart rate, arrhythmias and central nervous system excitation (headaches, insomnia, irritability, dizziness and lightheadedness).

Theophylline was the first amide/imide for which prodrugs were synthesized and were characterized for their ability to enhance the topical delivery of the parent drug from isopropyl myristate (IPM) vehicle [79,80].

**Figure 10:**
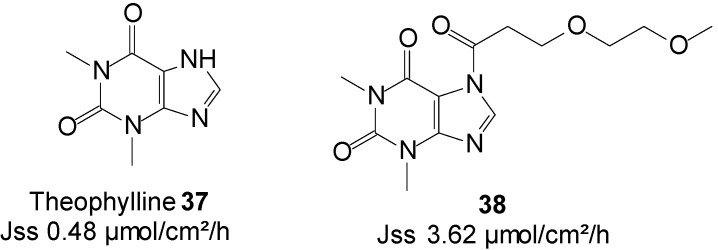
Theophylline and its best skin penetrant amide prodrug.

More recently, Majumdar* et al.* [81] investigated prodrugs of theophylline **37** by incorporating ethyleneoxy groups in the promoiety. The study resulted in an enhanced skin permeation rate of the drug. Prodrug **38** (Figure 10), featuring two EO units, achieved the highest delivery of total species through hairless mouse skin from IPM/PBS saturated solutions, which was more than seven times that of the parent, theophylline (3.62* vs.* 0.48 µmol/cm^2^/h).

### 5.9. Metoprolol Tartrate (β-Adrenergic Blocker)

Metoprolol tartrate, a β-selective adrenergic blocking agent, is a well-established drug for the treatment of angina pectoris, myocardial infarction, congestive heart failure and hypertension. Metoprolol reduces blood pressure by reducing cardiac output by slowing the heart rate, and is it a useful first line therapy in the treatment of mild to moderate essential hypertension. Its efficacy is, however, reduced by its extensive first-pass metabolism following oral administration. The transdermal route, by virtue of its capability to avoid the hepatic first-pass metabolic effect, is expected to achieve higher systemic bioavailability.

**Figure 11:**
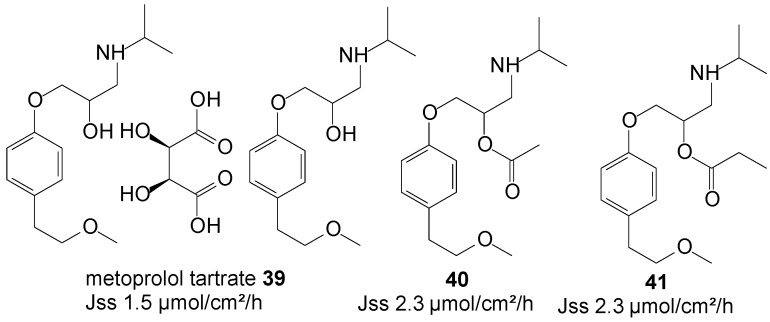
Metoprolol and its ester prodrugs.

Anroop* et al.* [82] conducted a comparative skin permeability study of metoprolol tartrate **39** and its ester prodrugs through passive permeation from 20% ethanol-acetate buffer pH 4 unsaturated solutions. The study resulted in both the acetate **40** and propionate **41** prodrugs (Figure 11) permeating the skin with similar flux, higher than the parent drug (2.3* vs.* 1.5 µmol/cm^2^/h). A marginal skin permeation enhancement was thus achieved using the prodrug approach in this study.

### 5.10. Captopril (Anti-Hypertensive)

Captopril is an angiotensin-converting enzyme inhibitor, primarily used for the treatment of hypertension. The use of captopril is commonly associated with rash and taste disturbances (metallic, or loss of taste). It also has a relatively poor pharmacokinetic profile, including poor oral bioavailability and a short half-life. Subsequently, it must be administered two or three times per day to reach therapeutic levels, which counteracts patient compliance. Transdermal drug delivery therefore has the potential to act as a viable alternative administration route for this drug by increasing its patient compliance profile.

**Figure 12:**
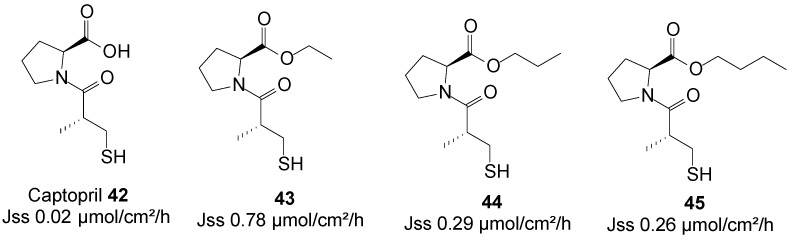
Captopril and its best *n*-alkyl ester prodrugs.

A series of *n*-alkyl carboxyl ester prodrugs of captopril **42** were synthesized and were subjected to percutaneous absorption in Franz diffusion cell experiments from saturated PBS pH 7.4 solutions using porcine skin. Captopril and its prodrugs crossed the skin relatively freely. The highest skin permeation fluxes were observed for the ethyl **43**, propyl **44** and butyl **45** ester prodrugs (Figure 12). Adoption of the prodrug strategy hence resulted in an increase in skin permeation of the parent captopril [83].

### 5.11. Hydroquinone (Anti-Melasma)

Hydroquinone **46** and salicylic acid **47** are drugs for treating melasma through the mechanisms of tyrosinase inhibition and chemical peeling, respectively. However, their frequency use is associated with skin irritation, which has limited use of both drugs. Salicylic acid is drug for peeling also known as an ultraviolet B sunscreen while hydroquinone is anti-melasma agent [84]. HQ in combination with peeling agents offers a synergistic therapy for treating melasma [85]. HQ is as a hydrophilic molecule with limited skin permeability and also poses some problems with storage instability and skin toxicity such as contact dermatitis and allergic dermatitis. To improve the efficacy and safety of melasma therapy, intense efforts has been devoted to enhance drug absorption and reduce toxic risk.

**Figure 13:**
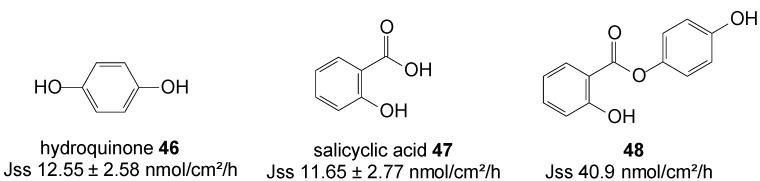
Structures of hydroquinone, salicylic acid and their best skin permeant ester co-drug **48**.

In order to promote cutaneous delivery of **46** and **47** for treating melasma, and to take advantage of the synergistic activity of skin lightening and chemical peeling combined in one unique entity, prodrugs of hydroquinone and salicylic acid were synthesized via covalent bonding (Figure 13) and their permeation through pig skin was assessed from 20% PEG400/PBC pH 7.4 saturated solutions. The 4-hydroxyphenyl 2-hydroxybenzoate prodrug or co-drug **48** permeated the skin with significantly higher flux compared to the individual parent drugs [86].

### 5.12. Haloperidol (Anti-Schizophrenic)

Haloperidol **49** continues to be a useful agent in the treatment of schizophrenia. The monthly intra-muscular administration of **49** in decanoate ester form proves to be valuable in reducing the problems of confused or agitated patients forgetting (or refusing) to take their medication. However, drawbacks associated to the administration of this ester by the intra-muscular route include local reactions and tenderness at the site of injection [87]. Furthermore, parenteral depot preparations require a healthcare professional to administer them, and short of removing the depot site by surgery, it is not possible to terminate treatment once it has commenced. Haloperidol delivered transdermally, and therefore avoiding the GI route, could among others be used to combat nausea and vomiting associated with opiate therapy in palliative care.

**Figure 14:**
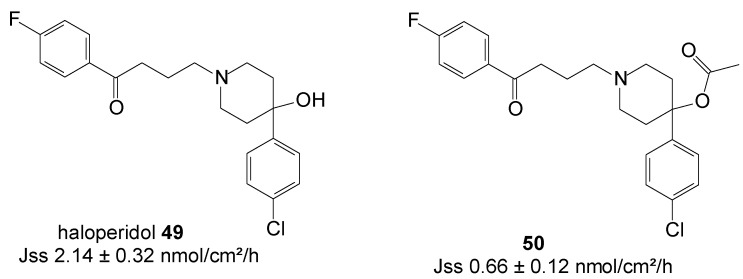
Structures of haloperidol and its ethyl ester prodrug.

Thus, a series of prodrugs including ethyl, propyl, butyl, octyl and decyl *O*-acyl esters of haloperidol were synthesized and their permeation across guinea pig skin were evaluated from aqueous PBS pH 7.4 saturated solutions. Cetrimide was included in the receptor phase as solubilizing agent but did not significantly alter the barrier properties of the membranes. As the prodrug ester chain length increased, the flux of the prodrugs decreased. The ethyl ester progrug **50** (Figure 14) was the best penetrant among all prodrugs. However, the permeation of the parent drug, **49**, across guinea pig skin was found to be greater than all its derivatives. Thus, no percutaneous absorption was enhanced by adopting the prodrug strategy in this study [88].

The physiochemical data, together with the skin permeation fluxes of all parent drugs and their best skin penetrant prodrugs, are summarized in Table 1.

**Table 1:** Physiochemical data and skin permeation fluxes of all parent drugs and best percutaneous penetrant derivatives.

Drug/Prodrugs	Therapeutic Category	M_W_ (g/mol)	S_w_ (mM) _PBS pH 7.4_	Log D _n-octanol/PBS pH 7.4_	Flux (nmol/cm^2^h)	Vehicle/Donor Phase Condition	Skin Penetration Enhancement	Ref.
Zidovudine 1 *	ARVs	267.2	95.4	−0.7 ± 0.04	8.55 ± 5.27	PBS pH 7.4/saturated	moderate	[44]
2		677.7	104.8	−1.1 ± 0.05	53.33 ± 46.51			
Stavudine 3 *		224.2	380.2	−1.1 ± 0.04	59.15 ± 23.28		no	[45]
4		414.4	nd	−0.7 ± 0.04	26.1 ± 22.23			
Lamivudine 5 *		229.3	82	−0.83 ± 0.06	4.23 ± 2.98 ^a^		no	[46]
6		285.32	18.0	1.9	0.046 ^a^			
7		331.34	25.8	−0.7 ± 0.01	2.07± 0.62 ^a^			[47]
8		375.40	nd	nd	0.66± 0.36 ^a^			
Cytarabine 9 *	Anticancer	243.2	738.8	−1.9 ± 0.02	3.7	PBS pH7.4/saturated	highly significant	[50]
10		341.4	14.53	0.91 ± 0.05	89.0			
11		345.3	230	−1.2 ± 0.03	8.3		moderate	[51]
12		313.3	48.46	−0.26 ± 0.00	22.2		significant	[52]
Morphine 13 *	Opioid Analgesic	285.3	>100	–1.13 ± 0.28	14.61 ± 4.08	PBC/unsaturated		[56]
14		341.4	30.1	1.75 ± 0.02	27.57 ± 4.57			
15		397.5	3.97	nd	72.45 ± 7.59	PBC/saturated	significant	
Naltrexone 16 *	Opioid Antagonist	341	0.26 ^h^	nd	2.5 ± 1.5	mineral oil/saturated	moderate	[61]
17		383	2.04 ^h^		15.6 ± 6.3			
18		397	4.41 ^h^		11.1 ± 3.7			
16 *		341	0.24 ^h^		3.06 ± 1.58		no	[62]
19		441	5.6 ^h^		3.73 ± 0.67			
16 *		341	0.24 ^h^		3.68 ± 2.37		moderate	[63]
20		412	1.01 ^h^		15.53 ± 11.26			
21		425	0.96 ^h^		17.44 ± 11.16		moderate	
Ketorolac 22 *	NSAIDs	255.27	70.87	–0.83 ± 0.08	2.12 ± 1.08	PBS pH 7.4/saturated	significant	[66]
23		395.50	35.64	2.15 ± 0.09	24.15 ± 3.50			
Ketorolac 22 *		255.27	19.1	1.14 ± 0.01	4.1	gel A	marginal	[67]
24		343.37	28.3	2.13 ± 0.02	6.71 ± 0.43	gel B		
Ketorolac 22 *		255.27	8205.2 ^b^	nd	3.24 ± 0.25	30% ethanol in PBC/saturated	moderate	[68]
25		311.2	172.8 ^b^	nd	8.21 ± 1.45			
Diclofenac 26 *		282.13	0.0041 ^c^	2.97 ^e^	2.57 ± 0.29	PBS pH 7.4/saturated	moderate	[69]
27		340.21	0.662 ^d^	1.57 ^f^	5.54 ± 0.78			
6-Meo-2-naphthylacetic acid 28		216.24	63.34	0.23 ± 0.02	1.44 ± 0.2	PBS pH 7.4/saturated	moderate	[71]
29		342.4	21.58	0.23 ± 0.01	6.80 ± 0.3			
30		356.5	20.09	0.22 ± 0.02	9.96 ± 0.7		moderate	
31		370.5	6.20	0.53 ± 0.08	16.10 ± 1.5		significant	
Apomorphine 32 *	Anti-Parkinsonian	267.3	nd	2.0 ± 0.1 ^g^	4.19 ± 1.03	Water/saturated	significant	[72]
33		407.51		1.6 ± 0.03 ^g^	51.46 ± 5.19			
Bupropion 34 *	Antidepressant	239.75	54.2 ^h^	nd	316.2 ± 15.6	mineral oil/saturated	no	[77]
35		255.74	3.9 ^h^		27.1 ± 4.2		moderate	
36		354.88	16.4 ^h^		72.5 ± 7.6			
Theophylline 37 *	Cardiovascular agent	180.17	0.3 ^i^	−2.14 ^j^	0.48 ^a^	IPM/saturated	moderate	[80]
38		326	2.6 ^i^	−1.52 ^j^	3.62 ^a^			
Metoprolol tartrate 39 *	β-Adrenergic blocker	684.82	>1000 ^k^	nd	1.5 ± 0.17 ^a^	20% ethanol-acetate buffer pH 4/unsaturated	marginal	[81]
40		309.41	8.3 ^k^		2.3 ± 0.17 ^a^			
41		323.43	5.9 ^k^		2.3 ± 0.17 ^a^			
Captopril 42 *	Anti-hypertensive	217.29	66.7		0.02 ^a^	PBS pH 7.4/saturated	highly significant	[82]
43		245.29	35.6		0.78 ^a^			
44		259.29	18.3		0.29 ^a^		significant	
45		273.29	18.0		0.26 ^a^		significant	
Hydroquinone 46 *	Anti-melasma	110.11	>50,000 ^l^	1.63 ± 0.01	12.55 ± 2.58	20% PEG400/PBC pH 7.4/saturated	moderate	[85]
48		230.22	2425.4 ^l^	3.77 ±0.05	40.86 ± 9.31			
Haloperidol 49 *	anti-schizophrenic	375.86	60.2 ^m^	nd	2.136 ± 0.316	PBS/saturated	no	[87]
50		417.90	10.8 ^m^		0.664 ± 0.123			

*: Parent drugs; ^a^: flux expressed in µmol/cm^2^ h; ^b^: Solubility in 30% ethanol; ^c^: Solubility in 5% PG (propylene glycol) in PBS pH 2.5; ^d^: Solubility in 5% PG in water; ^e^: Partition coefficient (octanol/5% PG PBS pH 2.5); ^f^: Partition coefficient (octanol/5% PG water); ^g^: Partition coefficient (octanol/water); ^h^: Solubility in mineral oil; ^i^: Solubility in IPM (isopropyl myristate), ^j^ partition coefficient (IMP/PBS pH 4.0); ^k^: Solubility in pH 4 acetate buffer; Gel A: carbomer (1%), triethanolamine (15%), **22** (15%), distilled water (83%); Gel B: carbomer (1%), triethanolamine (15%), **24** (15%), distilled water (83%); nd (not determined); PBC (phosphate buffer citrated); ^l^: Solubility (nmol/mL) in 20% PEG400/PBS pH 7.4 buffer; ^m^: Solubility (nmol/mL) in PBS.

The majority of derivatives listed in Table 1 demonstrated enhanced skin permeation fluxes in comparison with their respective parent drugs, regardless of the vehicle used. This is a further indicative that the vehicle-type is not a primary factor dictating percutaneous absorption of drugs, as previously reported [89]. The compounds also had molecular weights and partition coefficients in the recommended ranges (Sections 5.2.2. and 5.2.3.), which is in accordance early reports that these two factors are keys to the enhancement of skin permeation of drugs [89].

## 6. Conclusions

Research studies in recent years support the prodrug strategy as a viable approach which may be utilized to enhance skin permeation of therapeutic agents. However, in the design of a derivative that is intended to enter the systemic circulation through the skin, it is a prerequisite to ensure that the key physicochemical parameters of that compound, such as molecular weight and partition coefficient, are within the recommended ranges. These parameters are dependent of the promoiety linked to the parent scaffold. Therefore, the choice of the promoiety appears crucial in order to obtain optimum delivery percutaneous delivery of drug using the prodrug strategy.
